# Synthesis, Structure and Dielectric Characteristics of Tellurovanadate Glasses Containing Bismuth Oxide

**DOI:** 10.3390/ma18225239

**Published:** 2025-11-19

**Authors:** Tina Tasheva, Stanislav Slavov

**Affiliations:** 1 Department of Silicate Technology, University of Chemical Technology and Metallurgy, 8 Kliment Ohridski Blvd, 1797 Sofia, Bulgaria; 2 Department of Mathematics, University of Chemical Technology and Metallurgy, 8 Kliment Ohridski Blvd, 1797 Sofia, Bulgaria; stanislavslavov@uctm.edu

**Keywords:** tellurite glasses, vanadate glasses, dielectric properties

## Abstract

Two series of glasses with compositions (90 − x)TeO_2_-xV_2_O_5_-10Bi_2_O_3_ and (80 − x)TeO_2_-xV_2_O_5_-20Bi_2_O_3_, where x = 20, 30, 40 mol %, were synthesized. Glasses were obtained by the melt-quenching technique. The molar volume (33.48–48.37 cm^3^/mol) and oxygen packing density (71.69–77.64 mol/cm^3^) were calculated based on experimental density measurements. Both parameters increase with the increase in V_2_O_5_ and the decrease in TeO_2_ content. Infrared spectra were recorded in the range of 2000–400 cm^−1^ and Raman spectra in the 90–1280 cm^−1^ range. The C1s, O1s, Te3d, V2p and Bi4f photoelectron lines were recorded. The dielectric characteristics of the glasses were measured by impedance spectroscopy at room temperature in the frequency range from 100 Hz to 1 MHz. The glasses in the studied system demonstrate a strong dependence on the composition, with the occasional addition of high contents of TeO_2_ and V_2_O_5_ leading to a significant change in the dielectric properties of the samples.

## 1. Introduction

Over the past several decades, significant advancements have taken place in the high-tech domain of glass materials exhibiting electronic conductivity. Among these, tellurite, vanadate, and bismuthate glasses have attracted scientific attention due to their distinctive structural and functional behavior. Tellurite glasses are widely acknowledged for their suitability in optical technologies, a result of their notable features such as a high refractive index, strong transmission in the infrared region, robust chemical stability, and a comparatively straightforward melting process. Meanwhile, vanadate and bismuthate glasses are especially compelling due to their inherent semiconducting and electrical properties [1,2,3,4,5,6,7].

Vanadate glasses primarily consist of VO_5_ and VO_4_ polyhedra. Structural variations arise from the incorporation of different metal oxides (M^n+^Oₘ), which either occupy interstitial positions between vanadate chains, affecting the V=O bond vibrations, or substitute directly into the vanadate framework to form V–O–M linkages. This interaction alters the local symmetry and bond strengths, causing shifts in vibrational frequencies. In glasses with increasing modifier content, VO_5_ units gradually transform into VO_4_ tetrahedra, signifying a shift from vanadyl-type structures toward metavanadate chain networks. Similar mechanisms apply to tellurite and bismuthate glasses, where the high polarizability and covalency of Te^4+^ and Bi^3+^ facilitate their integration into the glass matrix, influencing both the structural rigidity and electronic properties. These glass systems exhibit a complex balance between localized structural units and extended network connectivity, contributing to their desirable optical and semiconducting characteristics [8].

Tellurite glasses, primarily composed of TeO_2_, exhibit a distinctive structure dominated by TeO_4_ trigonal bipyramids and TeO_3_ trigonal pyramids. The TeO_4_ units form the matrix of the glass network through corner-sharing with oxygen atoms, while TeO_3_ units typically emerge as a result of modifier addition or non-bridging oxygen formation. The structural flexibility between these units contributes to the glasses’ low phonon energy, high refractive index, and excellent infrared transmittance. The presence of lone-pair electrons on Te^4+^ ions plays a critical role in shaping the glass structure, introducing asymmetry and influencing the electronic polarizability. These features not only facilitate the incorporation of various functional dopants but also make tellurite glasses particularly attractive for applications in nonlinear optics, infrared photonics, and optical amplifiers. Furthermore, their relatively low melting temperatures and high chemical durability enhance their processability and suitability for advanced photonic device fabrication [9].

The inclusion of Bi_2_O_3_ in glass matrices significantly influences their structural and optical characteristics, primarily due to the large atomic mass and high electronic polarizability of Bi^3+^ ions. These factors lead to an elevated refractive index, which enhances the material’s suitability for use in photonic systems. From a structural standpoint, Bi_2_O_3_ can participate in the network as both a former and a modifier, facilitating the formation of BiO_6_ coordination units. This incorporation tends to disrupt the connectivity of the glass network, increasing the population of non-bridging oxygens and thereby reducing the degree of polymerization. Such changes are evident in FTIR spectral features and are typically correlated with a narrowing of the optical band gap, which allows for broader absorption in the visible and near-infrared regions. Additionally, the introduction of Bi_2_O_3_ lowers the phonon energy of the glass, which contributes to improved infrared transparency—an essential property for mid-IR photonic and laser applications. The presence of stereochemically active lone-pair electrons on Bi^3+^ may also enhance the nonlinear optical response of the glass. Beyond these optical benefits, Bi_2_O_3_ also imparts improved thermal stability and higher density, characteristics that are advantageous for applications in optoelectronic devices, protective optical components, and infrared transmission systems [10].

The aim of this study is to synthesize and characterize glasses within the TeO_2_–V_2_O_5_–Bi_2_O_3_ system, with particular emphasis on elucidating the influence of structural units such as TeO_4_/TeO_3+1_/TeO_3_, VO_5_/VO_4_, and BiO_6_ on the dielectric behavior. The novelty of this work lies in establishing clear correlations between the local structural configurations and the dielectric response of these multicomponent glasses, providing new insights into the structure–property relationships governing their functional performance.

## 2. Materials and Methods

Two series with compositions: Series 1: (90 − x)TeO_2_-xV_2_O_5_-10Bi_2_O_3_, where x = 10, 20, 30, 40, 50 mol %, and Series 2: (80 − x)TeO_2_-xV_2_O_5_-20Bi_2_O_3_, where x = 20, 30, 40 mol % were synthesized (Table 1). The initial reagents TeO_2_ (Alfa Aesar (Thermo scientific), ThermoFisher, Kandel, Germany, 99.99%), Bi_2_O_3_ (Acros Organics BV (Thermo Fisher Scientific), Geel, Belgium, 99.7%) and V_2_O_5_ (Alfa Aesar (Thermo scientific), ThermoFisher, Kandel, Germany 99.2%) were homogenized in an agate mortar, after which the resulting batches were melted at 950 °C for 20 min in an electric furnace. The melts were stirred several times during heating and then melt-quenched and pressed to a thickness of 1~2 mm. The experimental density of the glasses was measured using the Archimedes principle, using a Mettler analytical balance Toledo New Classic ME 104 (Mettler Toledo, Greifensee, Switzerland), equipped with a kit for determining the density of solid samples and distilled water as an immersion liquid. For each composition, the density is measured at least ten times in at least three different samples. From the density results obtained, the molar volume and oxygen packet density were calculated. Infrared spectra were recorded in the 4000–400 cm^−1^ range using the Varian 600-IR FT-IR spectrometer (Melbourne, Australia). The samples for these measurements were prepared in the form of KBr disks. The accuracy of absorption maxima is ±3 cm^−1^. Raman spectra were measured with the Raman Renishaw inVia microscope (Glocestershire, UK) with Leica DM2700 M (Leica Microsystems CMS GmbH, Wetzlar, Germany). The 633 nm line of a helium-neon laser was used for excitation. To avoid local overheating, the laser power used has been reduced to 3.2 mW. An X50 lens was used to focus the laser beam and collect the scattered light in a backscatter configuration. The diameter of the illuminated spot on the surface of the specimen is about 3 μm. The studied spectral range is 90–1280 cm^−1^. The accuracy of the wavenumber is 1 cm^−1^. The photoelectron spectra were recorded on an ESCALAB MK II X-RAY photoelectron spectrometer (Thermoscientific, VgScientific, East Grinstead, England) with a non-monochromatic X-ray source Al under a vacuum above 10^−7^ Pa at a 45-degree take-off angle and a total instrumental resolution of 1 eV. The bonding energies (BE) were determined using the C 1s line (of extra carbon) as a reference with an energy of 285.0 eV. The accuracy of the measured 18 19 connection energy is 0.2 eV. C 1s, O 1s, Te 3d, V 2p and Bi 4f photoelectron lines were recorded, and exploratory scanning was performed. The surface concentrations of the constituent elements were calculated from the peak area after subtracting the Shirley-type background. The dielectric characteristics were measured with the Zanner IM6 Impedance spectrometer (Zahner-Elektrik GmbH & Co. KG, Kronach, Germany) equipped with a standard sample holder with a two-electrode method. The samples were polished and plane-parallel, and silver electrodes were applied. Measurements of all samples were taken in the frequency range of 100 Hz–1 MHz at room temperature.

## 3. Results

### 3.1. Density, Molar Volume, Oxygen Packing Density

The results of the density measurements are presented in Table 1, Column 7. Based on the measured density, the molar volume (V_m_) was calculated using the following equation:

V_m_ = M_i_/d_i_ = (∑(x_i_·M_i_))/d_i_ (1)

where M_i_ is the molar mass of the glass, d_i_ is the density, x_i_ is the molar fraction of each component i, of the corresponding glass [11].

The oxygen packet density (OPD) of the glasses was determined by:

OPD = 1000·N_O2−_·d_i_/M_i_, (2)

where N_O2−_ is the number of oxygens per one molecule of glass [11].

**Figure 1:**
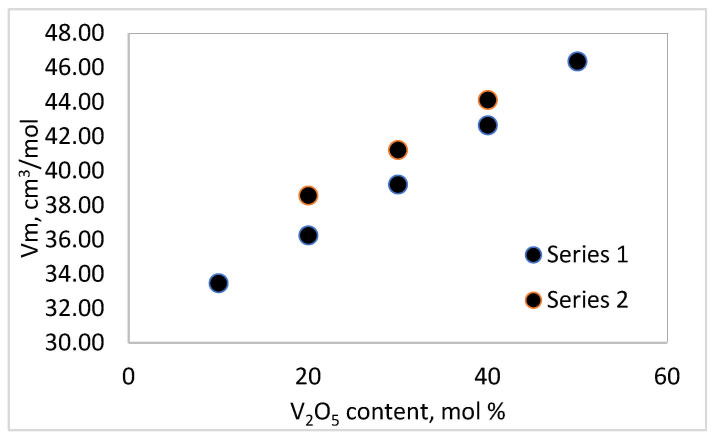
Molar volume (V_m_) as a function of the composition of the glasses in the TeO_2_.V_2_O_5_.Bi_2_O_3_ system.

**Figure 2:**
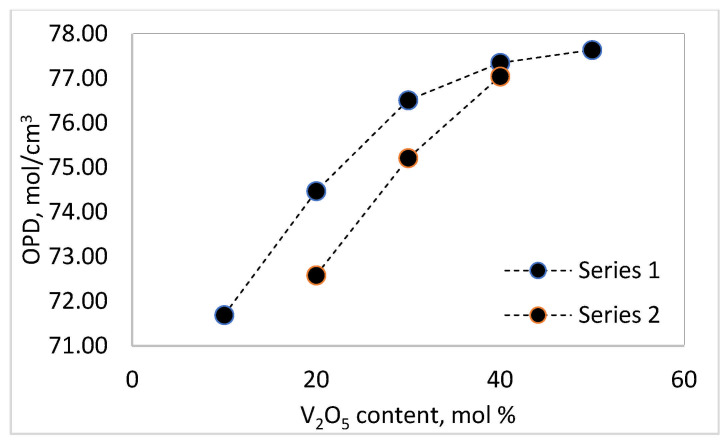
Oxygen packing density (OPD) as a function of the composition of the glasses in the TeO_2_.V_2_O_5_.Bi_2_O_3_ system.

Figure 1 and Figure 2 trace the relationships between the molar volume and oxygen packing density as a function of composition in the TeO_2_-V_2_O_5_-Bi_2_O_3_ system. The results show that the density decreases with an increase in the content of V_2_O_5_ and a decrease in the amount of TeO_2_, as the Series 2 glasses (20 mol % Bi_2_O_3_) have higher density values than those of Series 1 (10 mol % Bi_2_O_3_).

### 3.2. FT-IR Spectroscopy

**Figure 3:**
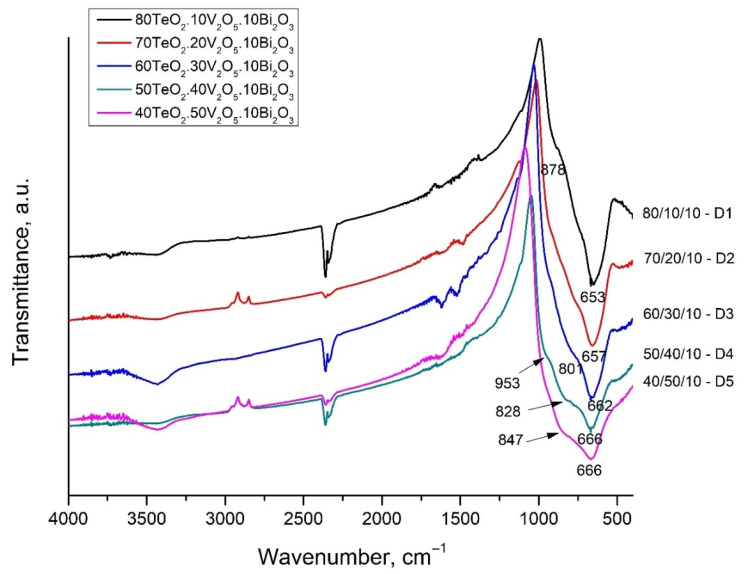
FT-IR spectra of the glasses of Series 1 (90 − x)TeO_2_.xV_2_O_5_.10Bi_2_O_3_ (x = 10, 20, 30, 40, 50 mol%).

**Figure 4:**
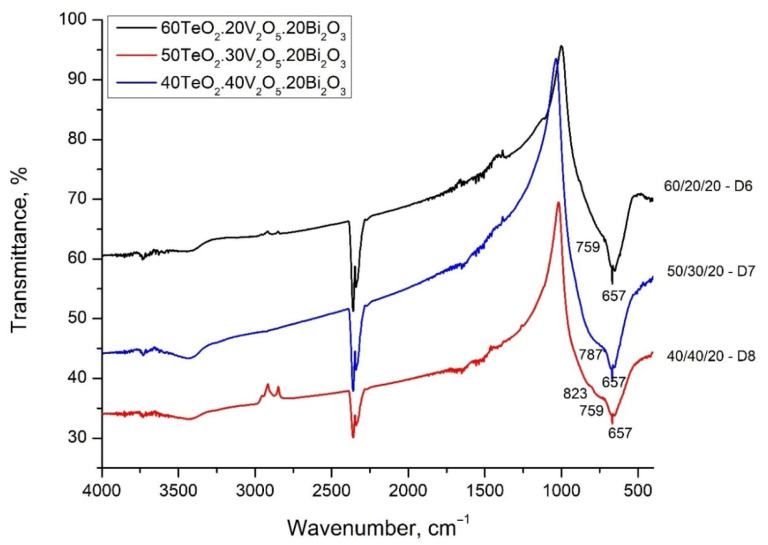
FT-IR spectra of the glasses of Series 2 (80 − x)TeO_2_.xV_2_O_5_.20Bi_2_O_3_ (x = 20, 30, 40, mol%).

With the aim of determining the main structural units that build up the structure of the glasses, the IR spectra of the glasses were recorded. The IR spectra of the glasses of System 1 (90 − x)TeO_2_.xV_2_O_5_.10Bi_2_O_3_ (x = 10, 20, 30, 40, 50 mol%) and System 2 (80 − x)TeO_2_.xV_2_O-20Bi_2_O_3_ (x = 20, 30, 40 mol%) are presented in Figure 3 and Figure 4. The spectra are characterized by a broad, well-defined band at 653–666 cm^−1^, and arms at 759–787 cm^−1^, 823–878 cm^−1^, and about 953 cm^−1^. It is noteworthy that with an increase in the content of V_2_O_5_, the appearance of an arm in the high-frequency region for both systems is observed, and the band at 653 cm^−1^ shifts slightly to 666 cm^−1^ in System 1 and does not change its position in System 2. Table 2 shows the position of the IR bands and their assignments.

### 3.3. Raman Spectroscopy

**Figure 5:**
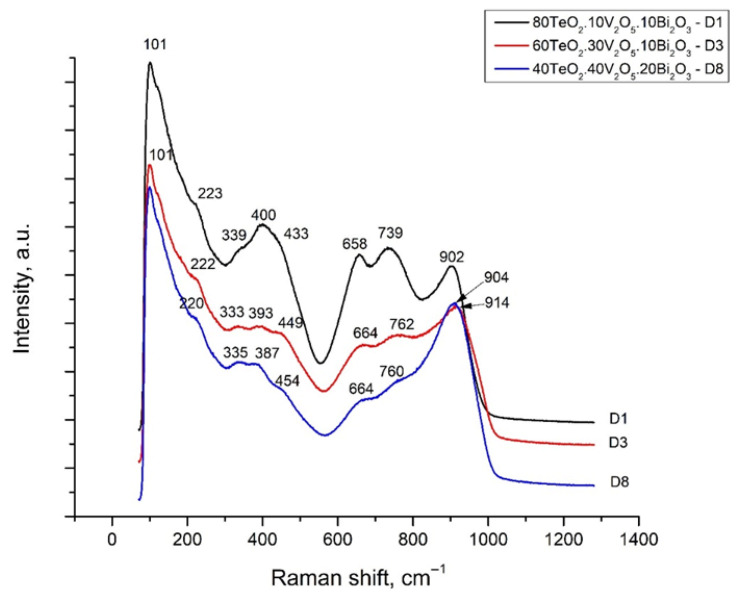
Raman spectra of glasses with compositions: 80TeO_2_.10V_2_O_5_.10Bi_2_O_3_, 60TeO_2_.30V_2_O_5_.10Bi_2_O_3_ and 40TeO_2_.40V_2_O_5_.20Bi_2_O_3_.

Raman spectra of three of the obtained glasses were recorded: 80TeO_2_.10V_2_O_5_.10Bi_2_O_3_—D1, 60TeO_2_.30V_2_O_5_.10Bi_2_O_3_—D3 and 40TeO_2_.40V_2_O_5_.20Bi_2_O_3_—D8 (Figure 5).

### 3.4. X-Ray Photoelectron Spectroscopy

**Figure 6:**
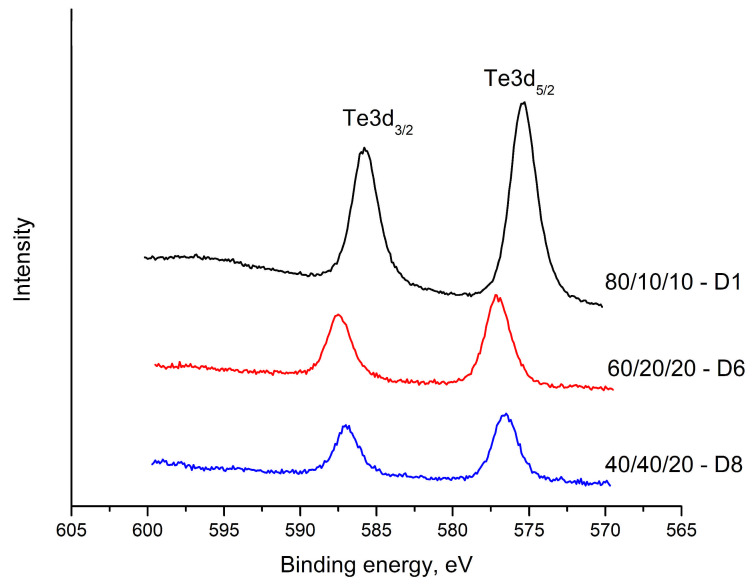
XPS spectra of glasses in the TeO_2_-V_2_O_5_-Bi_2_O_3_ system. Te3d spectra.

**Figure 7:**
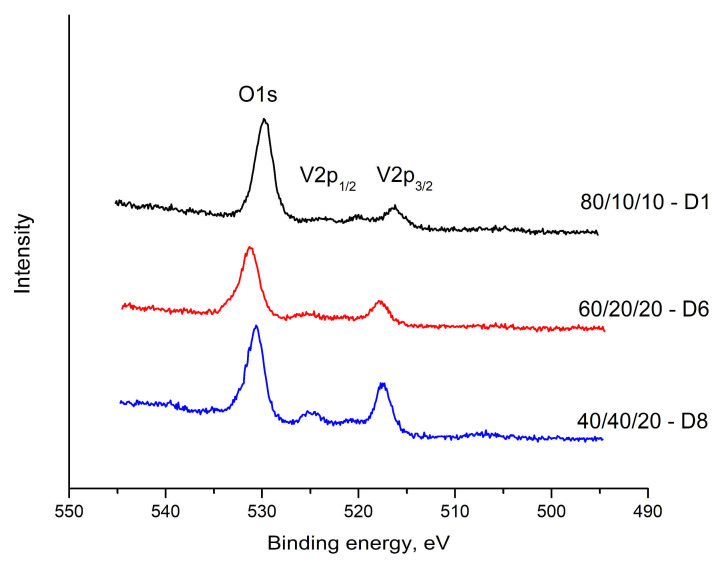
XPS spectra of glasses in the TeO_2_-V_2_O_5_-Bi_2_O_3_ system. O1s and V2p spectra.

**Figure 8:**
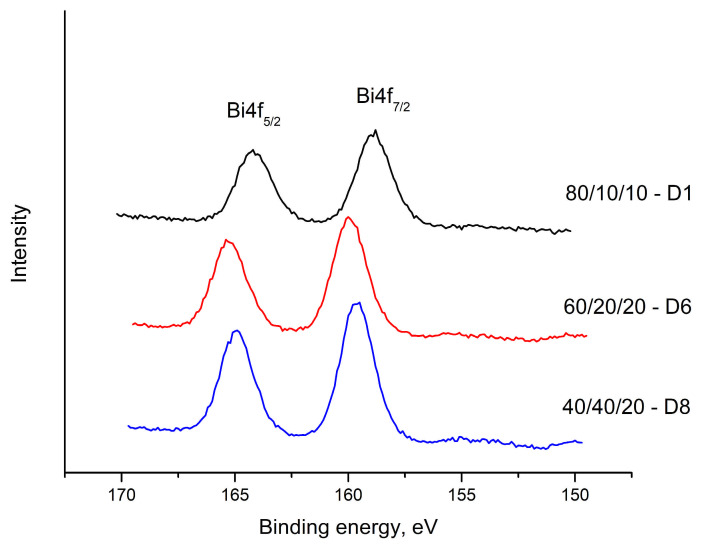
XPS spectra of glasses in the TeO_2_-V_2_O_5_-Bi_2_O_3_ system. Bi4f spectra.

Figure 6, Figure 7 and Figure 8 show the results obtained for the Te 3d, O 1s, V 2p and Bi 4f electron spectra of glasses with composition: 80TeO_2_.10V_2_O_5_.10Bi_2_O_3_ (D1), 60TeO_2_.20V_2_O_5_.20Bi_2_O_3_ (D6) and 40TeO_2_.40V_2_O_5_.20Bi_2_O_3_ (D8), in Table 3, the compositions and binding energies of Bi4f_7/2_, Bi4f_5/2_, V2p_3/2_, V2p_1/2_, O1s, Te3d_5/2_ and Te3d_3/2_ electrons are given, and in Table 4, their concentrations.

### 3.5. Dielectric Characteristics

**Figure 9:**
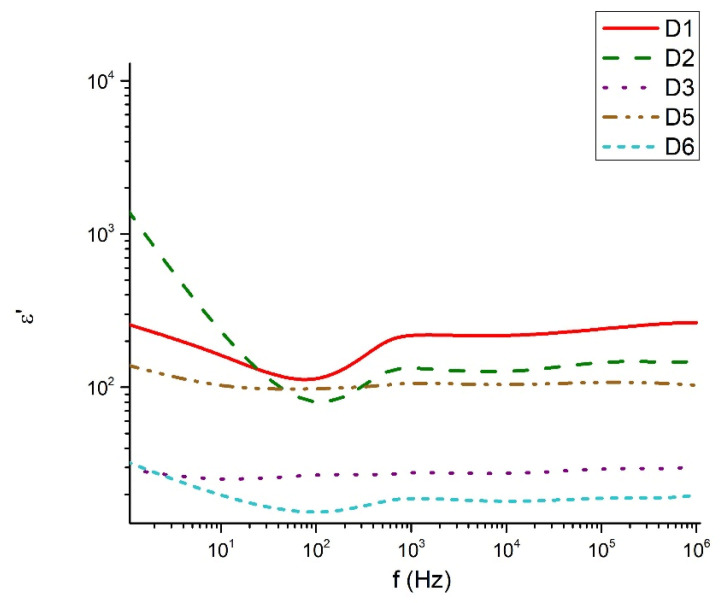
Real part of the dielectric permittivity as a function of the frequency.

**Figure 10:**
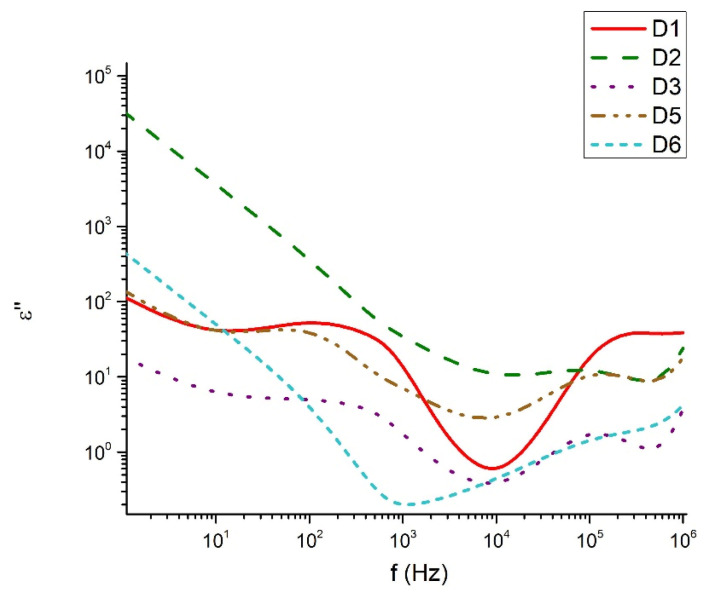
Imaginary part of the dielectric permittivity as a function of the frequency.

**Figure 11:**
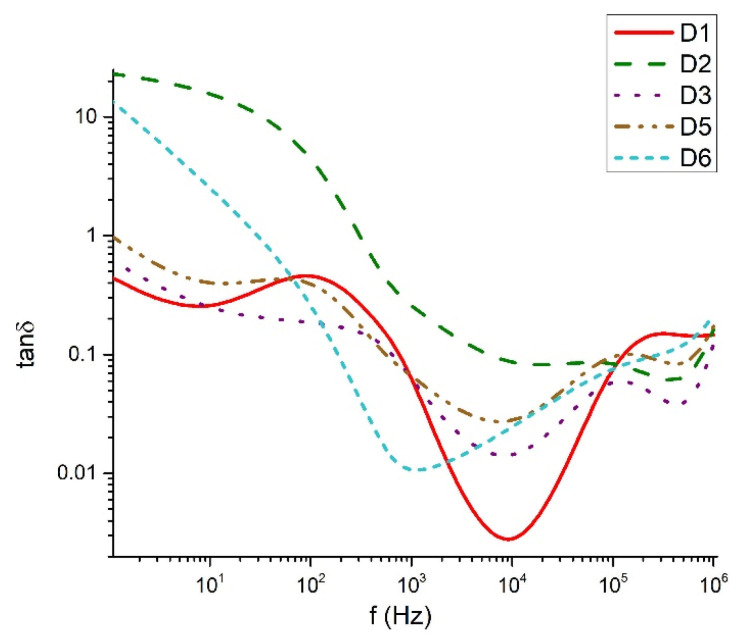
Dielectric losses as a function of the frequency.

**Figure 12:**
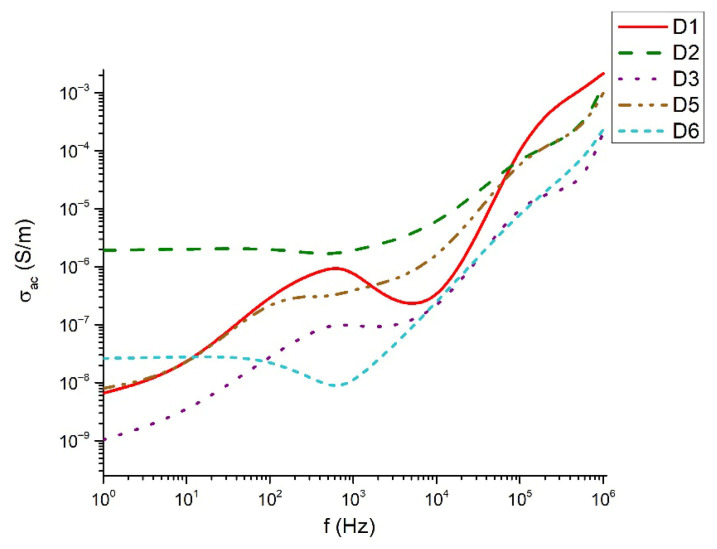
Conductivity as a function of the frequency.

Figure 9, Figure 10, Figure 11 and Figure 12 show the frequency dependencies of the dielectric permittivity, dielectric losses and conductivity of the glasses synthesized by us in the TeO_2_-V_2_O_5_-Bi_2_O_3_ system. The real part of the dielectric permittivity ranges from 20 to 300 (compositions 80TeO_2_-10V_2_O_5_-10Bi_2_O_3_ (D1), 40TeO_2_-50V_2_O_5_-10Bi_2_O_3_ (D5) and 60TeO_2_-20V_2_O_5_-20Bi_2_O_3_ (D6), depending on the degree of polarizability of the structural groups that make up the respective glass. The compositions mentioned above show a relatively weak frequency dependence of the real part of the dielectric permittivity (Figure 9). In different compositions, energy absorption varies according to frequency (Figure 10 and Figure 11). It is noteworthy that the compositions 80TeO_2_-10V_2_O_5_-10Bi_2_O_3_ (D1) and 40TeO_2_-50V_2_O_5_-10Bi_2_O_3_ (D5) from Series 1, have very low levels of dielectric losses and stable behavior of the real part of the dielectric permittivity in the entire studied frequency range. The conductivity of all samples is up to 0.002 S/m in the entire interval studied (Figure 12).

**Figure 13:**
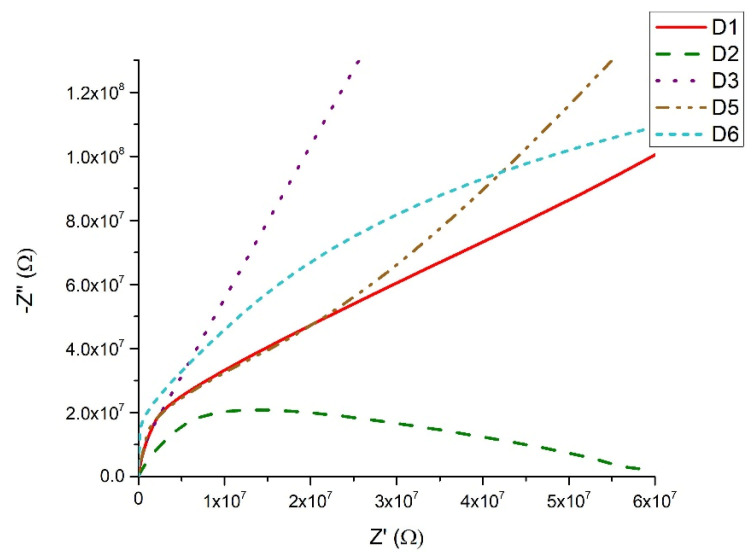
Nyquist plots for different resistance ranges of the samples.

Nyquist plots for different resistance ranges in Figure 13 of the samples show a semicircle for samples 80TeO_2_-10V_2_O_5_-10Bi_2_O_3_ (D1), 70TeO_2_-20V_2_O_5_-10Bi_2_O_3_ (D2), 60TeO_2_-20V_2_O_5_-20Bi_2_O_3_ (D6). The Nyquist plot contains overlapping semicircles, with the low-frequency part corresponding to the interface response and the high-frequency part to the bulk response. For samples 60TeO_2_-30V_2_O_5_-10Bi_2_O_3_ (D3) and 40TeO_2_-50V_2_O_5_-10Bi_2_O_3_ (D5) the semicircle is not complete, due to the limitation of the upper measurement limit of 1 MHz.

## 4. Discussion

The results obtained for the density of the samples are consistent with the fact that the atomic mass of V (50.94 u.) is lower than that of Te (127.6 u.) and Bi (208.98 u.), and the densities of pure TeO_2_, V_2_O_5_ and Bi_2_O_3_ are 5.67 g/cm^3^, 3.36 g/cm^3^ and 8.9 g/cm^3^, respectively. The molar volume and the oxygen packing density increase with the increase in the content of V_2_O_5_ and the decrease in TeO_2_. The molar volume, V_m_, expresses the free space and formation of non-bridging oxygens (NBOs) in the glass structure, while the oxygen packet density (OPD) is sensitive to the tightness of the structure as a whole. An increase in molar volume implies an increase in free space and the formation of non-bridging oxygens (NBOs) in the glass structure, and an increase in oxygen packet density is due to the formation of a stronger and highly cross-linked network, resulting in a more tightly packed amorphous network [11].

The analysis of the IR spectra was made based on the characteristic vibrations of metal-oxygen units found in various binary and ternary crystalline and amorphous materials with similar composition.

Dimitrov et al. [12] investigated the structure of the V_2_O_5_-Bi_2_O_3_ binary system by IR spectroscopy. They found that with an increase in the content of Bi_2_O_3_, the characteristic band at 1020 cm^−1^, associated with the vibration of the double V=O bond of VO_5_, the trigonal bipyramid disappears. This can be explained by the mechanism proposed by Dimitriev et al. [8]. According to them, Bi^3+^ cations directly attack the V=O bond as a result, of which the polyhedron is transformed into a VO_4_ group. The appearance of a band at 950 cm^−1^ is attributed to the vibrations of free VO_2_ groups of VO_4_ tetrahedra forming V-O-V bridges. The bands of these V-O-V bridges appear in the region of 840–820 cm^−1^. The absorption bands observed in the 760–740 cm^−1^ region are assigned to the triply degenerate bending vibration νₑ(F) of isolated VO_4_ tetrahedra, where each VO_4_ unit exists as a distinct, non-bridging structural entity within the glass network [12].

Dimitriev et al. [13] investigated the IR spectra of 2TeO_2_.V_2_O_5_ (2TV) as crystal and glass and found that the band at 950 cm^−1^ is associated with the valence vibration of the double non-bridge V=O bonds in VO_5_ trigonal bipyramids, and the band at 680–685 cm^−1^ in the spectra of TeO_2_-V_2_O_5_ glasses can be attributed to the valence asymmetric vibrations νdasTeO_3_ of TeO_3_ groups. For comparison, the band associated with the vibrations of the Te-O bond from the TeO_4_ polyhedron in pure tellurite glass is located at 654 cm^−1^ [14].

The observed displacement of the band at 653–666 cm^−1^ can be explained by the transformation of the tellurite groups. The band at 653 cm^−1^ in composition 80TeO_2_.10V_2_O_5_.10Bi_2_O_3_ is associated with asymmetric valence vibrations of symmetric TeO_4_ trigonal bipyramids and asymmetric valence vibrations of deformed TeO_4_ groups (TeO_3+1_), ν_as_TeO_2ax_. The last groups are referred to as TeO_3+1_, i.e., the tellurite atom forms one bridge, two non-bridge bonds, thus presenting itself as a TeO_3_ trigonal pyramid, but there is also a fourth bond, which is weak and strongly elongated. This structural group (TeO_3+1_) is also distinguished by very high oxygen polarizability and optical basicity [12]. Dimitrov and Komatsu [15,16] found that optical basicity in tellurite groups decreases in the order TeO_3+1_ > TeO_4_ > TeO_3_. With a decrease in the content of TeO_2_ and an increase in the content of V_2_O_5_, a significantly larger amount of symmetrical TeO_4_ trigonal bipyramids is formed compared to the deformed TeO_3+1_, as well as symmetrical TeO_3_ trigonal pyramids.

Glasses with a low V_2_O_5_ content do not show a pronounced band, but two barely noticeable arms, which become better pronounced with increasing V_2_O_5_ content—at about 800–847 cm^−1^ and at 953 cm^−1^. The band at 953 cm^−1^ could be assigned to the vibrations of free VO_2_ groups of VO_4_ tetrahedra forming V-O-V bridges. The band of these V-O-V bridges appear in the region of 840–820 cm^−1^. The bands at about 760 cm^−1^ are attributed to the triple-degenerate valence vibrations of isolated VO_4_.

Tagiara et al. [14] investigated the close order of binary and three-component tellurite glasses. In the spectrum of TeO_2_ glass, the presence of a strong band at 660 cm^−1^, a wide band at 700–850 cm^−1^ and low-frequency vibrations at 440 and 490 cm^−1^ are observed. The band at 700–850 cm^−1^ corresponds to the valence vibrations of the Te-O-Te bridges connecting the TeO_4_ trigonal bipyramids, as well as the vibration of the TeO_4_ trigonal bipyramid itself, while the deformation vibrations of O-Te-O and Te-O-Te are active at about 440 and 490 cm^−1^, respectively [14].

In the spectrum of glass with a composition of 80TeO_2_.10V_2_O_5_.10Bi_2_O_3_—D1, this band is strongly pronounced and even more intense than the strip at 660 cm^−1^ (TeO_4_ trigonal bipyramids). With an increase in the content of V_2_O_5_, the weak, elongated bond of the TeO_3+1_ polyhedra breaks and isolated TeO_3_ trigonal pyramids are formed and the position of the band shifts to about 760 cm^−1^. The intensity of all stripes associated with the vibrations of tellurite polyhedra and bonds decreases with increasing V_2_O_5_ content.

The band at 902–914 cm^−1^ in the Raman spectra of the glasses is attributed to the valence vibrations of V-O-V by VO_4_ tetrahedra [16]. The double degenerate bending vibration of the VO_4_ group is active at 333–339 cm^−1^ [17].

Figure 6 shows the electron spectra in the region of 600–570 eV. This area includes the doublets attributed to the Te 3d_5/2_ and Te3d_3/2_ electrons in the spectra. A chemical shift in the binding energies of 575.43–577.08 eV for Te 3d_5/2_ and from 585.86–587.47 eV for Te 3d_3/2_ was observed, with energy differences between the two peaks of 10.43 to 10.39 eV, respectively. The tellurite ion shows no change in its oxidation state. The observed chemical shift is probably due to the change in the tellurite polyhedron.

A single, well-defined peak is observed in the O1s region of the spectrum 531.20–528.59 eV (Figure 7). A change in intensity is noteworthy, as well as the presence of a chemical shift in this peak. The chemical displacement of the O1s binding energy is a function of the ionic-covalent nature of the metal-oxygen chemical bond. As the binding energy of O 1s decreases, oxygen polarizability and the basic character of oxides increase.

The peaks at 517.81–516.36 eV and at 525.23–523.24 eV in the electron spectrum of vanadium are attributed to V 2p_1/2_ and V 2p_3/2_, respectively. These binding energy values are assigned to V^5+^ and V^4+^, respectively (Figure 7).

In the region of 230–240 eV, Bi 4f spin-orbital cleavage is observed, as well as chemical displacement of the binding energies, from 159.69–158.87 eV to Bi 4f7/2 and from 164.22–164.95eV to Bi 4f5/2, with an energy difference of 4.53eV to 6.08eV, respectively. The intensity of the peaks decreases with an increase in the content of V_2_O_5_. This energy range is characteristic of Bi^3+^ (Figure 8).

The dielectric behavior of the studied tellurite–vanadate–bismuth glasses is closely linked to their structural features. Most samples exhibit similar real permittivity (~3.10^2^) across the measured frequency range, reflecting the dominant role of the tellurite network in establishing the base dielectric response. However, the compositions 60TeO_2_-30V_2_O_5_-10Bi_2_O_3_ (D3) and 50TeO_2_-40V_2_O_5_-10Bi_2_O_3_ (D4) show elevated permittivity values at low frequencies (<10 Hz). This enhancement is associated with the presence of highly polarized TeO_3+1_ units and VO_4_ tetrahedra, which may generate localized regions of uncompensated charges and contribute to increased dielectric losses. At high frequencies (>1 kHz), all samples exhibit a steady decrease in dielectric losses, remaining below 0.3. This behavior reflects the inability of large, highly polarizable structural units, such as TeO_4_ and TeO_3+1_ polyhedra, to follow rapid field oscillations, leading to reduced polarization and lower energy dissipation. In the intermediate frequency range (100 Hz–1 kHz), samples 60TeO_2_-30V_2_O_5_-10Bi_2_O_3_ (D3) and 60TeO_2_-20V_2_O_5_-20Bi_2_O_3_ (D6) show pronounced dielectric losses, indicating strong relaxation processes arising from structural heterogeneities, such as elongated Te–O bonds in TeO_3+1_ units and V–O–V linkages in VO_4_ tetrahedra. The variation in dielectric response can be rationalized in terms of network connectivity and polarizability. Glasses with higher V_2_O_5_ content (>40 mol%) form more symmetric TeO_4_ bipyramids and VO_4_ tetrahedra, increasing network rigidity and reducing the contribution of weakly bonded TeO_3+1_ units to low-frequency dielectric dispersion. In contrast, glasses with significant TeO_3+1_ populations (e.g., D1, D5) display reduced dielectric losses and more stable real permittivity due to the formation of isolated TeO_3_ trigonal pyramids, which limit charge accumulation and local polarization effects. The electrical conductivity data further support the structural interpretation. Conductivity remains negligible in the 100–2 × 10^4^ Hz range, consistent with the insulating nature of the cross-linked tellurite-vanadate network. Beyond this frequency, a sharp increase in conductivity occurs, reflecting the frequency-dependent polarization of TeO_n_ and VO_n_ units and the activation of localized charge transport associated with NBOs. Thus, the dielectric properties of these glasses are strongly governed by the interplay between structural units (TeO_4_, TeO_3+1_, VO_4_), network connectivity, and non-bridging oxygens. Tailoring the relative contents of TeO_2_, V_2_O_5_, and Bi_2_O_3_ allows for precise control over the polarization mechanisms, dielectric losses, and permittivity, providing a clear structural rationale for the observed frequency-dependent behavior.

## 5. Conclusions

Two series of glasses were synthesized: Series 1: (90 − x)TeO_2_-xV_2_O_5_-10Bi_2_O_3_, where x = 10, 20, 30, 40, 50 mol %, and Series 2: (80 − x)TeO_2_-xV_2_O_5–_20Bi_2_O_3_, where x = 20, 30, 40 mol %.

As the content of V_2_O_5_ and Bi_2_O_3_ increases, the number of non-bridge oxygens (NBOs) and the free space of the glasses increase. In parallel, with an increase in the content of V_2_O_5_, a stronger and cross-linked structure is obtained, and the addition of Bi_2_O_3_ worsens this cross-linking in glasses with a high content of TeO_2_ and a content of V_2_O_5_ below 40 mol %. Above 40 mol % V_2_O_5_, the addition of Bi_2_O_3_ does not indicate such deterioration of the amorphous network.

For glasses with a content of V_2_O_5_ up to 40 mol%, the dominant glass-forming agent is TeO_2_, and above 40 mol %, the dominant glass-forming agent is V_2_O_5_.

Glass with the composition 80TeO_2_.10V_2_O_5_.10Bi_2_O_3_ (D1) is composed of highly polarized, deformed TeO_4_ trigonal bipyramids, as well as isolated VO_4_ tetrahedra. The presence of Te^4+^, Bi^3+^ and V^4+^ ions is observed. A decrease in the TeO_2_ content leads to the formation of TeO_4_ trigonal bipyramids, as well as weakly polarized TeO_3_ trigonal pyramids.

Glasses made of highly polarized structural units are characterized by the highest values of frequency-dependent dielectric constant (real and imaginary part), dielectric loss and conductivity.

## Figures and Tables

**Table 1:** Compositions, molar mass (M), experimental density (d), molar volume (Vm), oxygen packing density (OPD).

	Sample	Composition, mol %			M, g/mol	d, g/cm^3^	Vm, cm^3^/mol	OPD, mol/cm^3^
		TeO_2_	V_2_O_5_	Bi_2_O_3_				
Series 1	D1	80	10	10	192.46	5.749	33.48	71.69
	D2	70	20	10	194.69	5.370	36.26	74.47
	D3	60	30	10	196.92	5.022	39.21	76.51
	D4	50	40	10	199.15	4.668	42.66	77.35
	D5	40	50	10	201.38	4.343	46.37	77.64
Series 2	D6	60	20	20	225.33	5.841	38.58	75.58
	D7	50	30	20	227.56	5.521	41.22	75.21
	D8	40	40	20	229.78	5.207	44.13	77.05

**Table 2:** Position of the IR bands and their assignments.

Position of the Band, ±3 cm^−1^	Assignments
~953	Valence vibrations of V-O from free VO_2_ of a VO_4_ tetrahedra, forming V-O-V bridges
847–828	Vibrations of V-O-V bridges from free VO_2_ groups
787–759	Triple degenerated valence vibrations of isolated VO_4_ tetrahedra
~666	Asymmetric valence vibrations of TeO_3_ trigonal pyramids, ν_d_^as^_TeOTe_, and asymmetric valence vibrations of axial Te-O bonds in TeO_4_ trigonal bipyramids (TeO_3+1_), ν^as^_TeO2ax_
~650	asymmetric valence vibrations of symmetric TeO_4_ trigonal bipyramids (TeO_4_), ν^as^_TeO2ax_. and asymmetric valence vibrations of deformed TeO_4_ groups (TeO_3+1_), ν^as^_TeO2ax_.
>500	Vibrations of deformed BiO_6_ groups

**Table 3:** Binding energies of Bi4f_7/2_, Bi4f_5/2_, V2p_3/2_, V2p_1/2_, O1s, Te3d_5/2_ and Te3d_3/2_.

	TeO_2_	V_2_O_5_	Bi_2_O_3_	Bi4f_7/2_	Bi4f_5/2_	V2p_3/2_		V2p_1/2_	O1s	Te3d_5/2_	Te3d_3/2_
D1	80	10	10	159.69	164.22	516.36	520.11	523.24	529.75	575.43	585.86
D6	60	20	20	159.21	165.29	517.81	521.48	525.23	531.20	577.08	587.47
D8	40	40	20	158.87	164.95	517.43	521.03	525.00	530.63	576.55	587.07

**Table 4:** Concentration of C 1s, Bi 4f, O 1s, V 2p, Te 3d.

	TeO_2_	V_2_O_5_	Bi_2_O_3_	C1s	Bi4f	O 1s	V 2p	Te3d
D1	80	10	10	36.75	4.89	36.76	3.45	18.15
D6	60	20	20	38.55	7.81	39.15	5.32	9.16
D8	40	40	20	41.27	7.55	37.16	7.37	6.65

## Data Availability

The original contributions presented in the study are included in the article, further inquiries can be directed to the corresponding author.
